# Monitoring of degradation of porous silicon photonic crystals using digital photography

**DOI:** 10.1186/1556-276X-9-410

**Published:** 2014-08-21

**Authors:** Maria Ariza-Avidad, Alejandra Nieto, Alfonso Salinas-Castillo, Luis F Capitan-Vallvey, Gordon M Miskelly, Michael J Sailor

**Affiliations:** 1 Department of Chemistry and Biochemistry, University of California, San Diego, 9500 Gilman Drive, La Jolla, California 92093-0358, USA; 2 Department of Analytical Chemistry, University of Granada, Faculty of Sciences, Avda. Fuentenueva s/n, Granada E-18071, Spain; 3 School of Chemical Sciences, The University of Auckland, Private Bag 92019, Auckland 1142, New Zealand

**Keywords:** Porous silicon, Photonic crystal, Degradation, Digital photography, Image processing, Hue color coordinate

## Abstract

We report the monitoring of porous silicon (pSi) degradation in aqueous solutions using a consumer-grade digital camera. To facilitate optical monitoring, the pSi samples were prepared as one-dimensional photonic crystals (rugate filters) by electrochemical etching of highly doped p-type Si wafers using a periodic etch waveform. Two pSi formulations, representing chemistries relevant for self-reporting drug delivery applications, were tested: freshly etched pSi (fpSi) and fpSi coated with the biodegradable polymer chitosan (pSi-ch). Accelerated degradation of the samples in an ethanol-containing pH 10 aqueous basic buffer was monitored *in situ* by digital imaging with a consumer-grade digital camera with simultaneous optical reflectance spectrophotometric point measurements. As the nanostructured porous silicon matrix dissolved, a hypsochromic shift in the wavelength of the rugate reflectance peak resulted in visible color changes from red to green. While the *H* coordinate in the hue, saturation, and value (HSV) color space calculated using the as-acquired photographs was a good monitor of degradation at short times (*t* < 100 min), it was not a useful monitor of sample degradation at longer times since it was influenced by reflections of the broad spectral output of the lamp as well as from the narrow rugate reflectance band. A monotonic relationship was observed between the wavelength of the rugate reflectance peak and an *H* parameter value calculated from the average red-green-blue (RGB) values of each image by first independently normalizing each channel (*R*, *G*, and *B*) using their maximum and minimum value over the time course of the degradation process. Spectrophotometric measurements and digital image analysis using this *H* parameter gave consistent relative stabilities of the samples as fpSi > pSi-ch.

## Background

Porous silicon (pSi) has proven to be a versatile material that is readily prepared and modified for use as chemical sensors or as a platform for drug delivery [1]. Porous silicon is suited for this latter role because pSi and the porous silica (pSi-o) formed upon oxidation are biocompatible and biodegradable. Porous silicon prepared with sinusoidal variations in the refractive index (termed rugate sensors) show one-dimensional photonic crystal behavior, with characteristic narrow-band rugate reflectance peaks that can be engineered to occur in the visible through infrared regions of the electromagnetic spectrum. The reflectance spectra of these sensors changes when analytes enter or leave the pores or if the pore walls are dissolved. The ability to place the peaks in the reflectance spectrum within the near infrared region of the electromagnetic spectrum allows direct monitoring through tissue [2-4] which has potential use for both biomonitoring and monitored drug release.

Most optical studies of porous silicon-based materials have used spectrophotometers with reflectance probes. The position of the wavelength of the maximum reflectance peak of a porous silicon-based photonic crystal can be an effective reporter of degradation due to oxidation and dissolution of the silicon matrix in aqueous media. Spectrophotometric measurement of the temporal evolution of the visible reflectance spectrum of pSi or pSi-o has been used to follow the dissolution process and the release of drugs trapped in the porous matrix [5,6].

A key challenge we are addressing is the development of efficient low-cost methods to extract relevant chemical information from the change in optical response of porous silicon and similar nanostructured sensor materials. The broad-band red-green-blue (RGB) filters in most color cameras are not optimal for measuring changes in porous silicon reflectivity. The complete optimization of such camera-chemical sensor combinations will require structuring the optical properties of the nanosensor material to best match the optical response of the camera, optimizing the illuminant, and development of efficient and selective data analysis algorithms. In this paper, our primary focus is on developing a simple single parameter to represent the change detected by a color camera as a porous silicon film degrades.

Colors, which are qualities representing human visual experiences [7,8], can be quantified by a number of methods or color spaces. Color spaces can be classified into four groups related via algebraic transformations: linear-light tri-stimulus, xy chromaticity, perceptually uniform, and hue-oriented [8]. All of the color spaces can be derived from the RGB information supplied by devices such as cameras and scanners. The hue, saturation, and value (HSV) color space used in this work represents the cognitive color information associated with a change in dominant wavelength of the observed signal in a single parameter, the hue coordinate *H*. The use of hue in optical sensor devices has been reported previously, especially in investigations of bitonal optical sensors and of thermochromic liquid crystal thermography. Thus, all relevant color information in digital images of bitonal sensors (sensors in which a chromophore changes into another chromophore with a different spectrum in the presence of a given analyte) is contained in the *H* coordinate [9,10]. These authors note that the *H* coordinate is simple to calculate, is easily obtained from commercial imaging devices, and shows little dependence on variations in color intensity or variations in brightness of illumination.

The reflectance spectra of the thermochromic liquid crystals used in thermography are similar to those of rugate porous silicon, having narrow reflectance peaks with width 30 to 40 nm [11,12]. These reflectance peaks can move over 100 nm to the blue as temperature increases. Thermochromic liquid crystal thermography often relies on a monotonic relationship between hue and temperature. However, several authors have noted that the measured hue is dependent on the illuminant used and is also impacted by background reflectance [11-13]. This can result, for example, in hue not being monotonic if a red-rich light such as a tungsten lamp is used. Anderson and Baughn noted that approaches such as subtracting the amount of light in each of the red, green, and blue channels observed at low temperature from all subsequent measurements and then calculating hue using these corrected values could give a monotonic *H* function for all the light sources they used [11,12]. They noted that a monotonic *H* function was also obtained if they adjusted the white balance of their measurements using the image data corresponding to the low-temperature liquid crystal rather than images of a ‘true gray’ [11]. The concept of deriving a hue-based function after modification of the raw intensity data has been extended further. Thus, Finlayson and Schaefer applied logarithmic preprocessing to obtain a hue parameter that was invariant to brightness and gamma [14], while van der Laak et al calculated absorbance for transmitted light microscopy images prior to determining a hue parameter [15].

There are additional complexities with analyzing digital images of rugate porous silicon compared to thermochromic liquid crystals because the reflectance peaks can be narrower (10 to 30 nm) and the reflectance peak intensities can change to a larger extent with wavelength, due to factors such as light absorption within the porous silicon layer or degradation of the porous layer.

In this work, we aimed to use a consumer-grade digital camera to monitor the degradation of freshly etched and modified pSi photonic crystals (rugate filters) rather than using a spectrophotometer. While this constrains the reflectance measurements to lie within the visible spectrum, measurement of the spectral changes of pSi by digital photography can enable monitoring of pSi degradation and drug delivery in non-laboratory settings. The use of digital photography for monitoring the degradation of pSi in aqueous media was validated by simultaneous spectrophotometric measurements of the pSi reflectance spectrum.

## Methods

### Preparation of freshly etched porous silicon chips (fpSi)

Porous silicon was prepared by anodic electrochemical etching of highly doped 0.95 mΩ cm p^++^-type (100)-oriented silicon wafers (Virginia Semiconductor, Fredericksburg, VA, USA) in a 3:1 (*v*/*v*) mixture of aqueous hydrofluoric acid (49%) and ethanol.

The fpSi samples were prepared in a Teflon etch cell that exposed 1.2 cm^2^ of the polished face of the Si wafer, which was contacted on the back side with a piece of Al foil. A platinum spiral was used as a counter-electrode. A rugate filter was generated using a current density modulated with 100 cycles of a sinusoidal waveform oscillating between 15 and 108 mA/cm^2^, with periods on the order of 6 s depending on the desired wavelength of maximum reflectivity. After etching, the fpSi samples were rinsed with ethanol and dried in a stream of nitrogen.

### Preparation of porous silicon coated with chitosan (pSi-ch)

A 1% chitosan solution was prepared by dissolving 10 mg chitosan from crab shells, 85% deacetylated (Sigma Aldrich, St. Louis, MO, USA) in 1 mL of 15% *v*/*v* aqueous acetic acid and stirring overnight. The fpSi sample was coated with chitosan by spin coating (Laurell WS-400B-6NPP-Lite, Laurell Technologies, North Wales, PA, USA) using 150 μL of chitosan solution at a final speed of 100 rpm for 10 min and then drying at room temperature under nitrogen. The sample was then placed under vacuum to evaporate the remaining solvent. After the deposition, the pSi-ch samples were heated at 70°C on a hot plate for 10 min to cause a small amount of polymer infiltration into the pores, and this resulted in a slight red shift in the rugate reflectance peak position.

### Instrumental procedures

The porosity and thickness of the porous silicon layers were estimated by the spectroscopic liquid infiltration method (SLIM), based on the measurement of the thin-film interference components of the reflectance spectra of the samples before and after infiltration of a liquid (ethanol) with known refractive index [16] by using an Ocean Optics USB-2000 spectrometer (Ocean Optics, Dunedin, FL, USA) configured for specular reflectance, working in back-reflection configuration in the range 400 to 1,000 nm. The reflectance spectra were recorded at five spots distributed across each sample in order to evaluate the homogeneity of each porous silicon sample. The values of the porosity and the thickness were determined by means of the two-component Bruggeman effective medium approximation [17]. The extent of chitosan infiltration into the porous silicon sample was also evaluated from the reflectance spectrum.

The freshly prepared (fpSi) and modified (pSi-ch) porous silicon samples were characterized using a Thermo Scientific Nicolet 6700 FTIR (Thermo Fisher Scientific, Waltham, MA, USA) with a Smart iTR diamond ATR fixture. The morphology of the porous silicon was measured by scanning electron microscopy (SEM) using a FEI XL30 SEM (FEI, Hillsboro, OR, USA) operating in secondary electron imaging mode. To avoid sample charging anomalies, the porous Si samples were metalized with a thin layer of gold prior to the SEM analysis. The pore size and the porosity oscillations of the rugate filter structure were evaluated with this analysis.

### Measurement of porous silicon degradation

**Figure 1:**
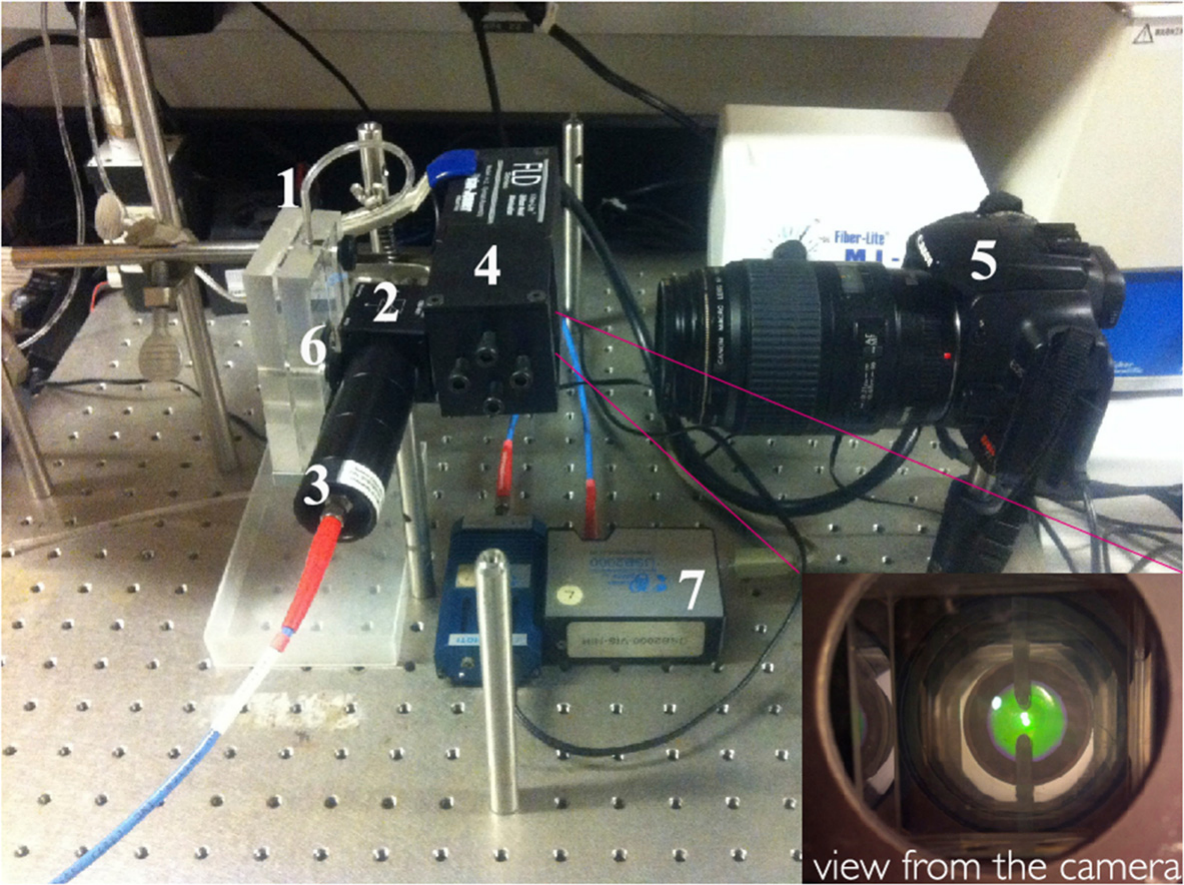
**Photograph of equipment for simultaneous acquisition of photographs and reflectance spectra.** 1 flow cell containing pSi sample, 2 beam splitter, 3 reflectance probe connected to fiber-optic spectrophotometer, 4 diffuse axial illuminator with tungsten light source, 5 camera, 6 pSi sample, and 7 spectrophotometer**.** Inset: image of the pSi sample as captured by the digital camera.

The pSi degradation was studied using a custom-designed transparent flow cell system with a total volume of 4.5 mL (including connections). The 1:1 (*v*/*v*) ethanol 0.5 M carbonate/borate buffer solution (pH 10) was flowed in at the bottom of the sample using a peristaltic pump at a rate of 10 μL/s and room temperature (20 ± 1°C). Ethanol was included in the buffer to improve the permeation of solution into the pores and reduce the formation of bubbles that could affect the subsequent image analysis.The degradation of the fpSi and pSi-ch samples was monitored by obtaining reflectance spectra (spectrophotometer) and photographs (digital camera) every 5 min through the front cover of the flow cell until after complete degradation had occurred (300 min). To obtain both measurements repeatably during the same experiment, the optical paths for the reflectance probe and the camera were located in front of the flow cell along the sample surface normal as is shown in Figure 1. The sample was illuminated by means of a diffuse axial illuminator coupled to a Fiber-lite MI-150 (Dolan Jenner, Boxborough, MA, USA) light source with an approximate color temperature of 3,000 K mounted between the flow cell and the camera. A beam splitter (Thorlabs CM1-BS2 Cube-Mounted Non-Polarizing Beamsplitter, 50:50, 0.7 to 1.1 μm; Newton, NJ, USA) between the diffuse axial illuminator and the flow cell also allowed measurement of the reflectance spectrum over 400 to 1,000 nm with the reflectance probe of a fiber optic spectrophotometer (Ocean Optics USB-2000-VIS-NIR). The reflectance probe was rigidly fixed to the beamsplitter via lens tubes containing a focusing lens.

The reflectance spectrum acquisition was controlled by Spectrasuite software (Ocean Optics, Inc.). The position of the rugate reflectance peak and the FFT of the portion of each reflectivity spectrum that displayed Fabry-Perot interference fringes were calculated using custom routines in Igor (Wavemetrics, Inc., Portland, OR, USA). The shifts in the position of the peaks in the FFT spectra indicate a change in effective optical thickness (EOT or 2 *nL* where *n* is the effective refractive index and *L* is the thickness of the layer) of the porous silicon samples.

Digital images were acquired with a Canon EOS 500D (Digital Rebel XTi; Canon, Ota, Tokyo, Japan) digital camera with an EF-S 60 mm *f*/2.8 macro lens. In order to use the camera as a colorimeter, the geometry of the imaging equipment was rigidly fixed and the flow cell was exposed to constant lighting. The camera settings were fixed at ISO 400, aperture value *f/*4.5, shutter speed 1/2 s, and white balance set for a tungsten light source. Canon EOS Utility software was used to remotely operate the camera from a computer and to transfer the jpg images from the camera to the computer.

### Image analysis

**Figure 2:**
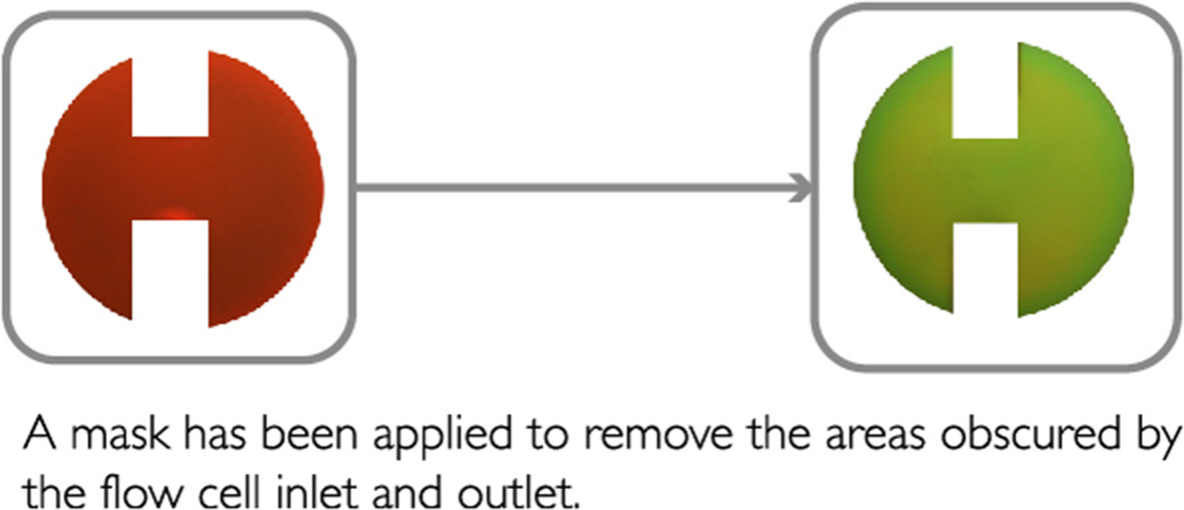
Images showing color change of pSi sample during degradation and mask used to select pixels for image analysis.

The jpg images were pre-processed using Photoshop CS5 (Adobe Systems, San Jose, CA, USA). First, a color curve balance correction for each image was made selecting as a reference point a portion of the silicon wafer that was not in contact with the buffer solution. Next, the portion of each image containing the pixels corresponding to the degrading porous silicon sample (ca. 1.2 × 10^5^ pixels) was defined using a mask, Figure 2. The average RGB values for these pixels were determined for each image. The *H* coordinate, or hue, [9] of the HSV (hue, saturation, and value) color space, was used to monitor the porous Si degradation since it represents the dominant color in one single parameter. The RGB values of the selected pixels in each image were processed with a set of scripts and functions developed in Matlab r2010b (The MathWorks Inc, Natick, MA, USA) to determine the *H* coordinate, which is defined as in Equation 1.

(1) $$\begin{array}{c} H=\left(\frac{G-B}{\max_{\text{min}}-\min_{\mathrm{channel}}}+0\right)\;.60;\;\mathrm{if}\;\max=R^{*}\\ H=\left(\frac{B-R}{\max_{\text{min}}-\min_{\mathrm{channel}}}+2\right)\;.60;\;\mathrm{if}\;\max=G\\ H=\left(\frac{R-G}{\max_{\text{min}}-\min_{\mathrm{channel}}}+4\right)\;.60;\;\mathrm{if}\;\max=B \end{array}$$

* if *H* less than 0, then add 360 to *H*.

The *H* coordinate in the HSV color space has a circular nature and so can be defined as an angle that varies between 0 and 360° [18]. However, because of the processing we have used prior to our *H* calculation, we report the values on a 0 to 1 scale. *H* values calculated by applying the above equations to the as-acquired images were not monotonic with time. A monotonic function was obtained in the following manner: The average RGB values for each image were normalized, with each channel being normalized independently using the maximum and minimum value for that channel observed during the degradation process. The *H* value of these processed values was then calculated.

## Results and discussion

### Characterization of porous Si

The different porous Si rugate samples had thicknesses in the range 20 to 25 μm and average porosities of 53 to 62%, and displayed a single narrow band between 581 and 603 nm in their visible reflectance spectra.

**Figure 3:**
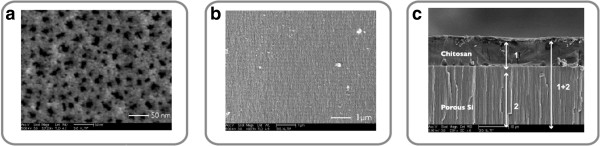
**SEM images of the porous silicon. (a)** Top view showing the pore openings in fpSi. **(b)** Partial cross-section showing the rugate modulations in porosity in fpSi. **(c)** Cross section of chitosan-coated porous silicon (pSi-ch).

**Figure 4:**
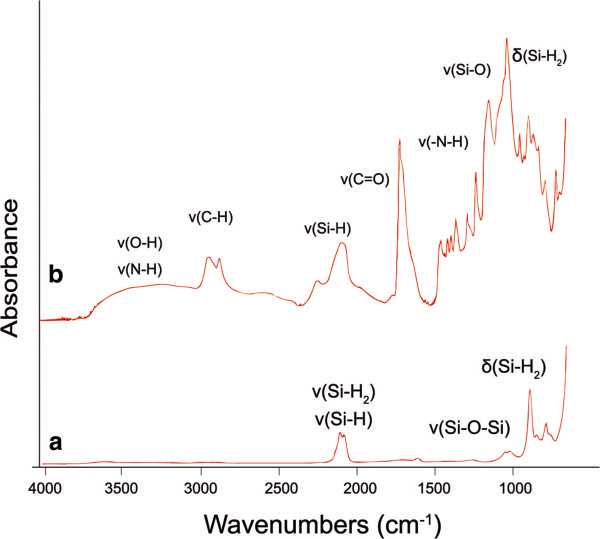
ATR-FTIR spectra of (a) freshly etched pSi (fpSi), (b) freshly etched pSi with a layer of chitosan (pSi-ch).

The freshly etched porous Si samples had the maximum reflectance peak centered at 593 nm (standard deviation 3.7 nm; *n* = 5). The thickness and porosity of fpSi were 22.8 μm (1.2 μm) and 53.4% (1.6%), respectively, both measured by SLIM. The average diameter of the pores was 20 nm as calculated from top surface SEM images (Figure 3a), and a channel-like mesoporous structure was observed in cross-sectional SEM images (Figure 3b). The ATR-FTIR spectrum of fpSi (Figure 4a) shows a band at 2,100 cm^-1^ due to the presence of Si-H_*x*_ groups (*x* 1 to 3) [19], a 905-cm^-1^ band assigned to the SiH_2_ scissor mode [20], and a 667-cm^-1^ band due to SiH wagging mode. The small band at 1,050 cm^-1^ due to Si-O stretching modes suggests a small amount of oxidation has occurred after etching [21].

**Figure 5:**
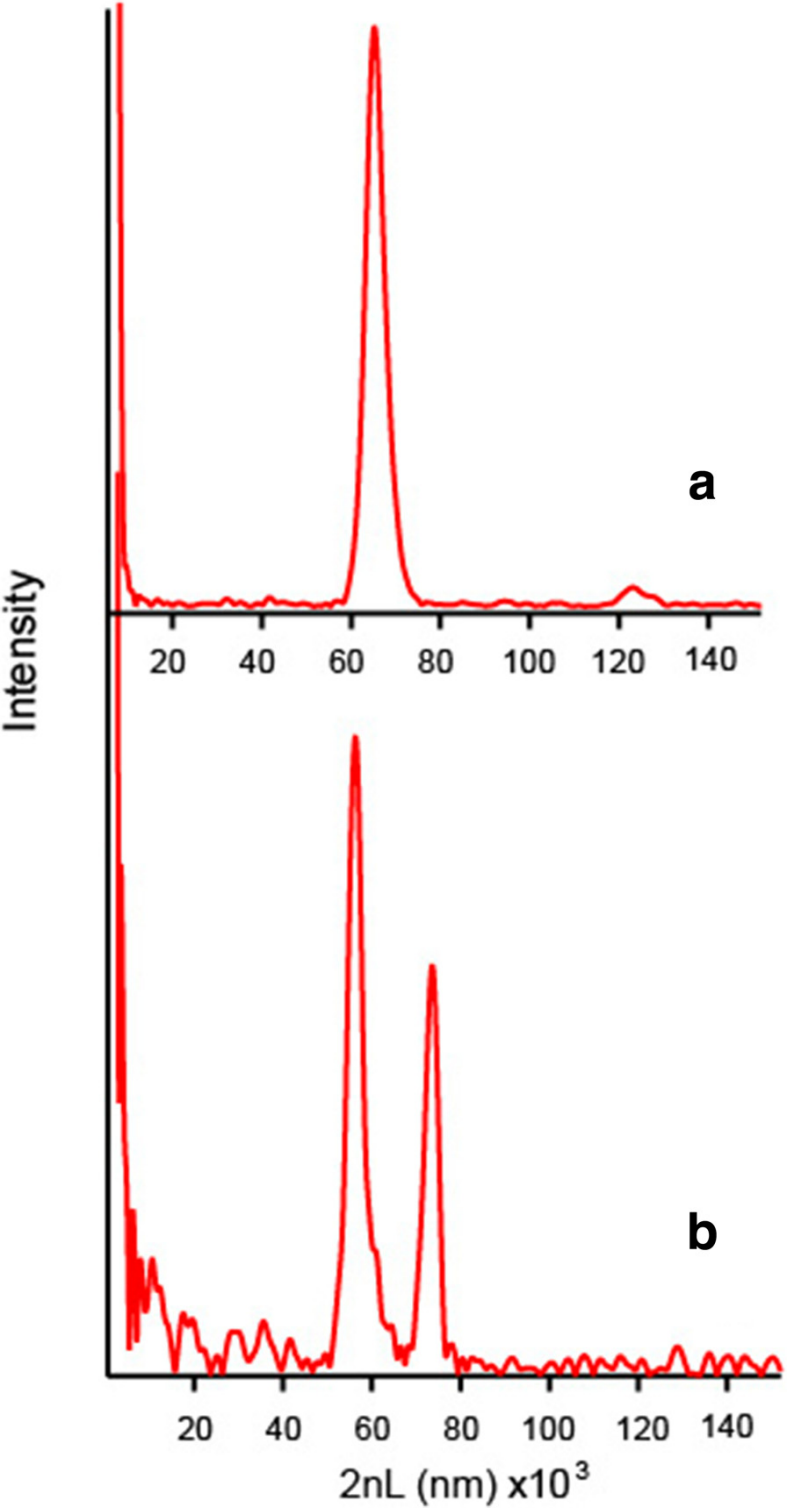
FFT of the visible reflectance spectrum obtained from pSi with (a) and without (b) a coating of chitosan.

Chitosan, a positively charged natural polysaccharide which is both biodegradable and biocompatible, was investigated as a protective coating for pSi due to its reported potential use in drug delivery studies [22]. A film of chitosan was deposited on the porous Si surface by spin coating. In order to evaluate the infiltration of the chitosan into the pores of the fpSi sample, cross-sectional SEM and reflectance spectra were compared before and after chitosan coating. The range of thickness achieved by spin coating was 8 to 12 μm according to SEM results, with the two well-defined separate layers suggesting the chitosan was mainly present as an adherent layer on top of the porous silicon (Figure 3c). More precise information about the extent of chitosan infiltration into pSi was obtained from reflectance spectra of the hybrid. The reflectance spectra of the fpSi samples coated with chitosan showed a red shift of 8 nm in the maximum of the rugate peak. However, analysis of the thin-film interference fringes which are also present in the reflectance spectrum allowed more detailed investigation of the changes to the pore filling. When chitosan is spin-coated onto the pSi surface and then warmed slightly, the chitosan forms an optically smooth film on top of the pSi layer, which leads to an additional Fabry-Pérot optical interference layer. Therefore, the FFT of the reflectance spectrum displays two major peaks (Figure 5). The position of the peak at an effective optical thickness (EOT) of 60.2 μm (EOT_2_ = 2*n*_2_*L*_2_, where *n*_2_ is the effective refractive index of the layer and *L*_2_ is its thickness) is slightly larger than the position of the corresponding peak observed in the FFT spectrum of the unmodified fpSi (59.7 μm). This peak is assigned to the pSi layer initially and to the pSi layer including a small amount of incorporated chitosan after modification. The second major peak in the FFT spectrum appears at an EOT of 77.4 μm (EOT_3_ = 2*n*_3_*L*_3_). This peak comes from interference between the top surface of the chitosan (the air/chitosan interface) and the bottom of the porous Si film (the bulk Si/porous Si interface). A third peak with EOT_1_ = 2*n*_1_*L*_1_ that is expected for the chitosan layer at 17.2 μm, according to the relationship EOT_1_ + EOT_2_ = EOT_3_, is not observable due to the small difference between chitosan and pSi refractive indexes [23]. These data indicate that chitosan does not significantly infiltrate the porous Si layer and are in agreement with the SEM images and the results from Pastor et al. who concluded that chitosan penetration into the inner structure of partially oxidized pSi is hindered [24]. Thus, the structure of pSi-ch samples consists of an array of porous reservoirs capped with a chitosan layer.

Upon loading of chitosan onto the fpSi, new bands appear in the FTIR spectrum (Figure 4b). The broad band at 3,350 cm^-1^ is assigned to both O-H and N-H stretching; the bands at 2,915 and 2,857 cm^-1^ are due to C-H stretching vibration modes, while the aliphatic CH_2_ bending appears at 1,453 cm^-1^ and the C = O stretching vibration mode appears at 1,710 cm^-1^. The intense band at 1,043 cm^-1^ has contributions from the C-O stretching mode in addition to Si-O stretching modes [5].

### Monitoring of porous silicon degradation

Hydride-terminated porous silicon undergoes degradation when immersed in aqueous solutions, with release of gaseous or soluble species, due to two processes: (1) oxidation of the silicon matrix to silica by water or various reactive oxygen species and (2) hydrolysis to soluble orthosilicic species [25]. This degradation hinders its use in some applications although controlled degradation is useful for applications such as drug delivery. Different strategies have been applied to improve the stability of porous silicon [26], such as oxidation of the surface under controlled conditions [27], derivatization forming Si-C bonds on the surface via different organic reactions [28,29], or covering the porous structure with protective polymeric films [5].

The degradation of porous silicon in aqueous solution depends on several factors, with pH being a key factor. In acidic or neutral aqueous media, the degradation proceeds slowly but in basic solutions, hydroxide reacts with both Si-H and Si-O surface species [1]. A pH 10 buffer solution that would lead to moderately rapid degradation of the porous Si samples (time for degradation <300 min) was selected for this study. Ethanol was added to the buffer to ensure wetting of the porous silicon layer and to reduce the formation of adherent gas bubbles on the samples.

**Figure 6:**
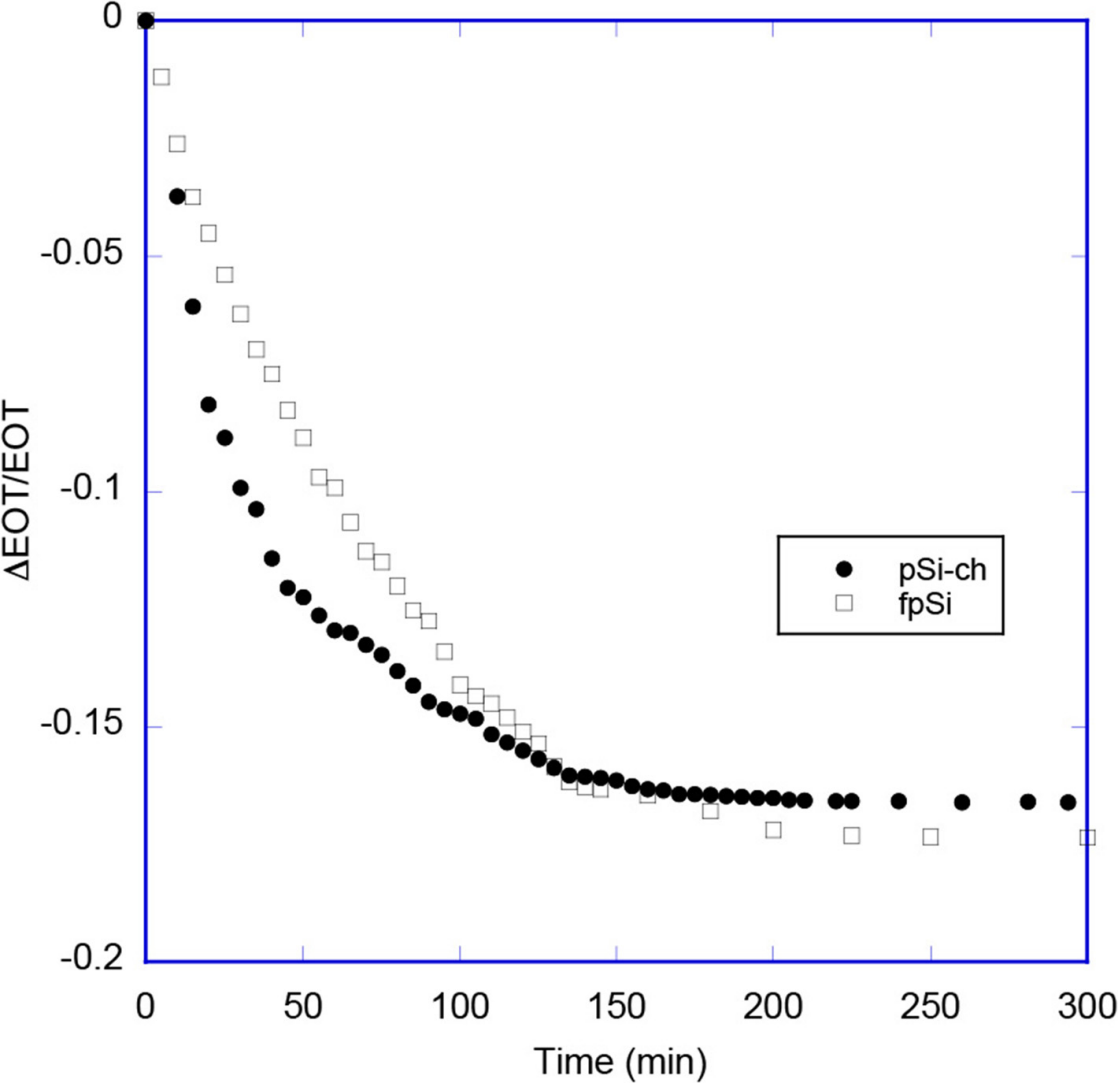
**EOT changes observed during the degradation of the two porous Si sample types.** Plots showing the relative change in the effective optical thickness (EOT) of the pSi samples as a function of time exposed to 1:1 (*v*/*v*) 0.5 M carbonate/borate buffer (pH 10), ethanol at 20 ± 1°C.

Porous Si rugate filters show characteristic reflectance spectra due to the periodic oscillations of porosity in the direction normal to the surface. Changes in the average refractive index of the porous silicon film due to infiltration of compounds into the pores or alteration of the porous silicon matrix modify the wavelength of maximum reflectance providing a useful method for sensing [30]. The oxidation of the porous silicon matrix to silica decreases the effective refractive index, which causes a hypsochromic shift in the position of the maximum reflectance peak in the spectrum, and the dissolution of the porous layer can both decrease the thickness of the layer and increase the porosity, both processes leading to a reduction in the effective optical thickness. Therefore, the shifts in the Fabry-Perot interference fringe pattern observed in the visible reflectance spectra and the wavelength of the rugate peak maximum can be used to measure and compare the stability of different porous Si samples. The effective optical thickness of porous silicon samples can be obtained in real time using a fast Fourier transform of the reflectance spectra [1,31]. One strategy to then compare the degradation of different porous Si surface samples in aqueous media involves calculating the relative change in effective optical thickness defined as

(2) $$\Delta \mathrm{EOT}/\mathrm{EO}T_{0}\text{\%}=\left(\mathrm{EOT}\;-\;\mathrm{EO}T_{0}\right)/\;\mathrm{EO}T_{0}\;\times \;100\text{\%}$$

where EOT_0_ is the value of EOT (Equation 2) measured when the porous Si surface is initially exposed to flowing buffer. The degradation of the pSi surface is then monitored by this relative decrease in optical thickness [32]. The degradation of the two porous Si sample types in the present study as measured by EOT changes is shown Figure 6. The data indicate that the stability of these samples decreases in the sequence: freshly etched porous Si > chitosan-coated pSi, since the initial rates of relative EOT change during the degradation are 0.217 and 0.37%/min, respectively. The degradation rate is higher for porous silicon coated by chitosan than for fresh pSi for the first 25 min, but there is a subsequent decrease in the degradation rate of the chitosan-coated sample so that at later times it degrades more slowly than fresh porous silicon, with relative EOT changes of 0.066 and 0.108%/min, respectively. The increased rate of degradation for the chitosan-coated porous silicon sample is in apparent contrast to the previously reported studies of chitosan-coated porous silicon, however, those studies used hydrosilylated porous silicon or oxidized porous silicon [5,23,24]. The increased degradation of pSi-ch compared even to freshly etched porous silicon may be due to the amines present in chitosan, since amines can increase the rate of porous silicon hydrolysis [33,34]. It also suggests that the chitosan layer contains cracks or fissures such that the aqueous solution readily infiltrates to the underlying fpSi layer.

The stability of the pSi samples as shown by the rates of change of the positions of the band maxima of the rugate reflectance bands of the samples during their degradation followed the same order as for the EOT measurements: freshly etched porous Si > chitosan-coated pSi, with the rates being 1.33 and 1.99 nm/min, respectively.

**Figure 7:**
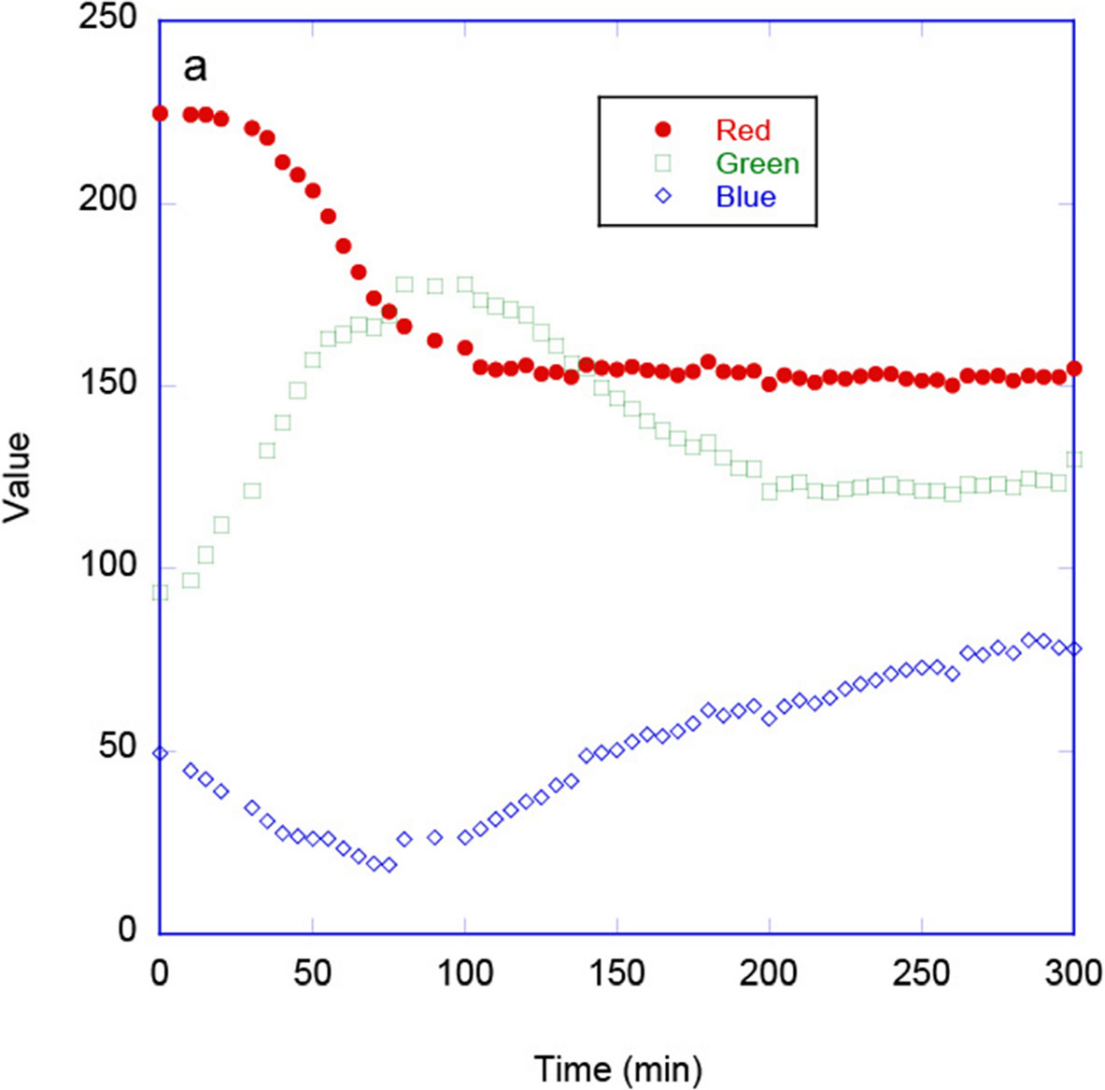
Plot showing the change in average RGB values from images of fp-Si as it degrades.

**Figure 8:**
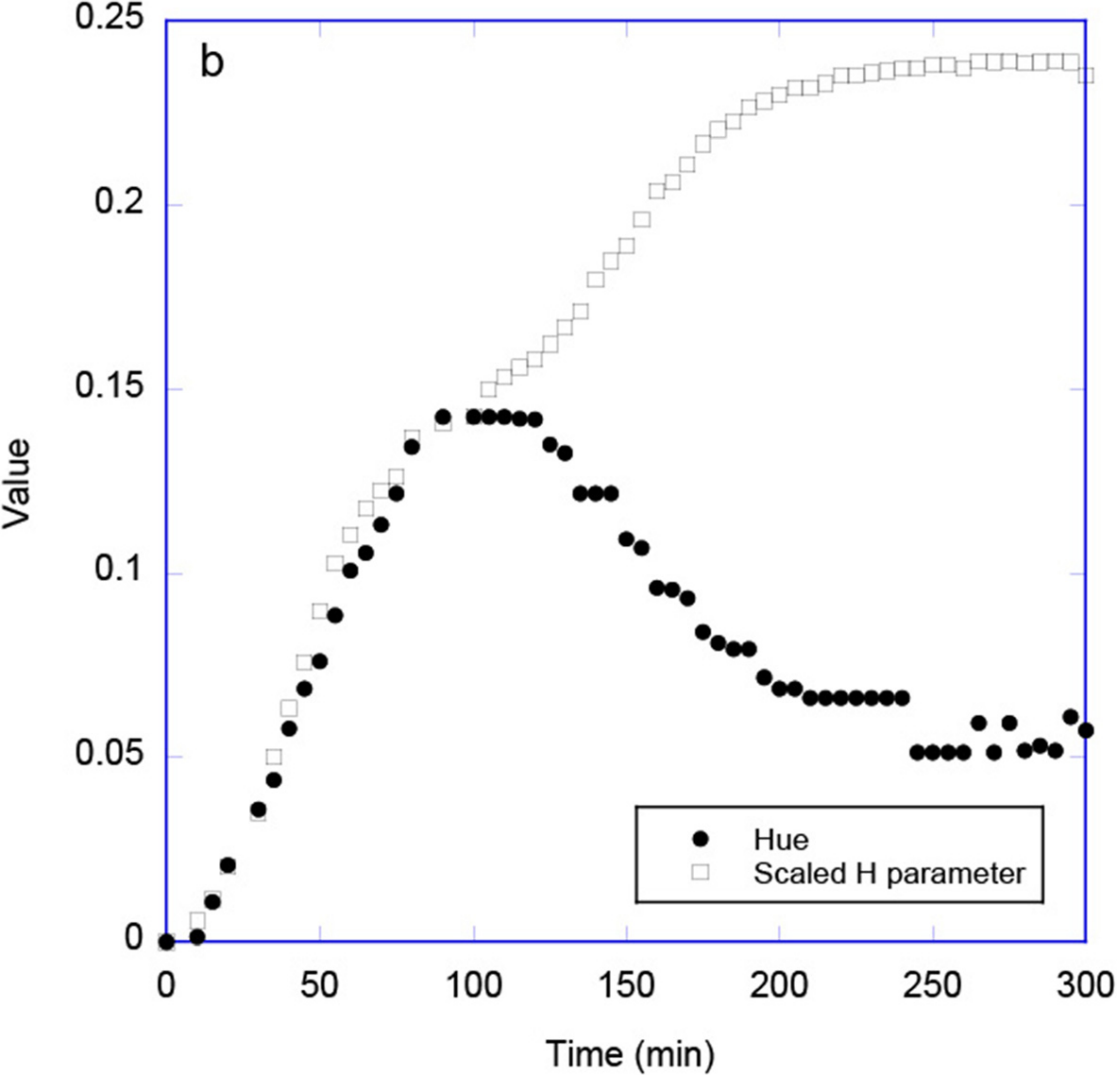
**Plot showing hue derived from as-acquired images and scaled *H*-parameter derived from pre-processed RGB values.** The *H* parameter has been scaled for this plot so that hue and the *H* parameter have the same numerical value at 100 min.

The degradation of porous Si, typically monitored by reflection or transmission measurements using a spectrophotometer, can also be monitored using digital photography if the degradation results in a perceived color change. Since previous studies have reported that the *H* coordinate of the HSV color space can provide a robust single parameter that corresponds to changes in the position of the main band in a reflectance spectrum of an optical sensor [9,10], we investigated whether this *H* coordinate could be used to monitor the shifts in wavelength and intensity of the narrow rugate reflectance band as porous silicon degrades. We initially investigated calculating the *H* coordinate for the as-acquired images, Figures 7 and 8. As the porous silicon degradation process occurred this *H* coordinate (hue) increased from ca. 0.033 to a maximum value of 0.18. These changes in the *H* coordinate values were manifested in a visible color change from red to green and a decrease and increase in the red and green channels of the images, respectively (Figure 7). Once all the pSi had dissolved, the mirror-like silicon wafer substrate was exposed. Reflection of the tungsten light source from this bare silicon surface was yellow as captured by the camera. This reflection from the substrate resulted in a *reduction* in the magnitude of the hue from ca. 0.18 to 0.11 at long times (at time >100 min), Figure 8.

Because of this non-monotonic behavior of hue, we investigated other functions of the red, green, and blue channels that might provide a measure of degradation over the whole time of the reaction. We found that pre-processing the data by taking the average red channel value for each image and normalizing it using the minimum and maximum observed average red values during the degradation process and doing the same for the other two channels and then applying Equation 1 to these normalized channels gave a suitable monotonic function, Figure 8. Since the value obtained does not correspond directly to the perceived color, we refer to it as the *H* parameter. As noted in the ‘Background,’ other authors have developed useful *H* parameters derived from HSV transformation of pre-processed data [11,12]. Our pre-processing is analogous to a combination of the background correction reported by Anderson and Baughn [11,12,14,15] followed by a white balance correction. The relative change in this *H* parameter was very similar to that for hue over the first 100 min of the degradation reaction, but at longer times the *H* parameter continued to increase in a monotonic manner in contrast to the behavior of the hue. Measurement of the color evolution using this *H* parameter confirmed the previously observed trend regarding the stability of the porous silicon samples towards degradation.

**Figure 9:**
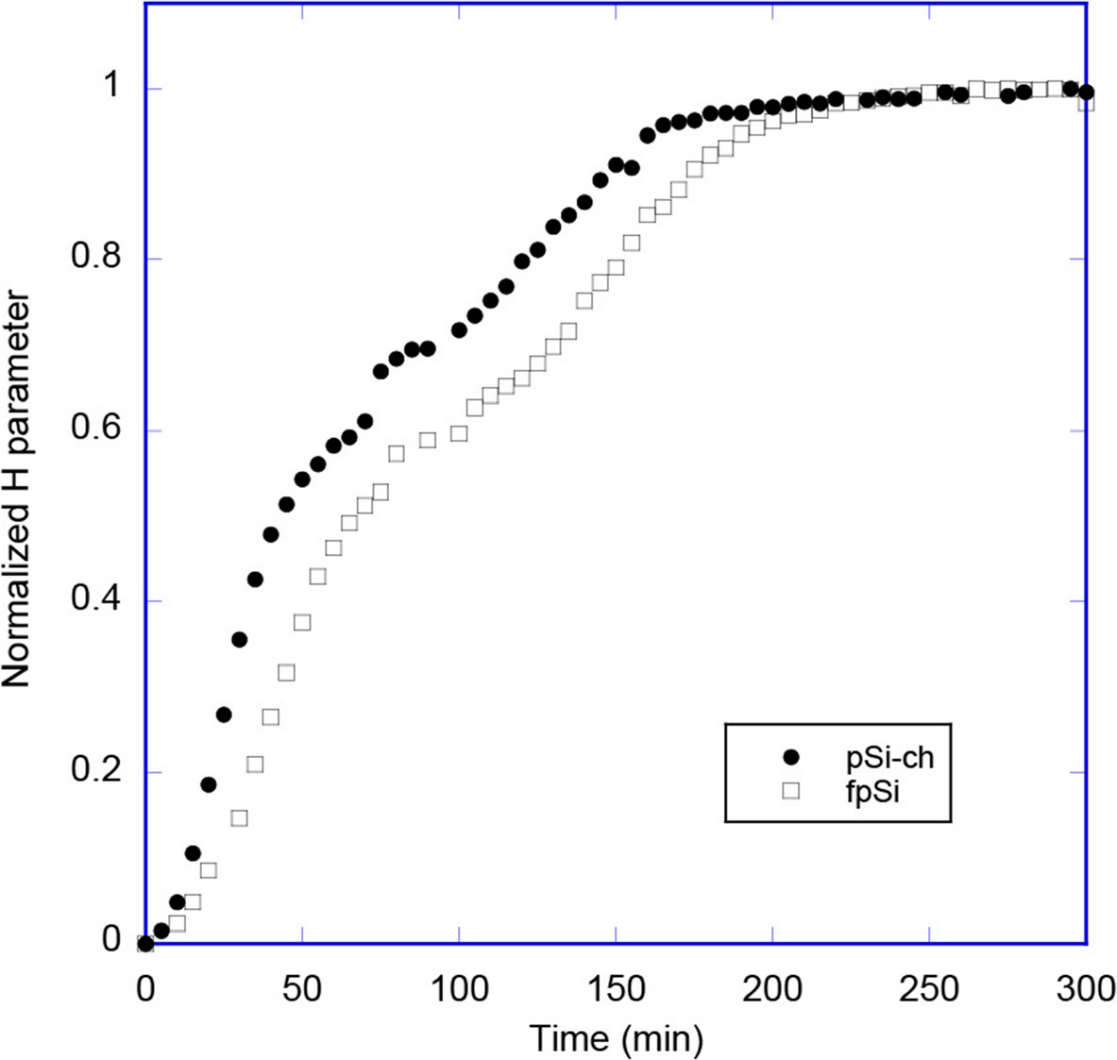
**Evolution of the normalized *H *parameter during the first 300 min for fpSi and pSi-ch.** The experimental conditions are as given for Figure 6.

We then used this *H* parameter to compare the degradation of the two porous silicon samples. Thus, Figure 9 shows a comparison of the normalized value ((*H* - *H*_initial_)/(*H*_max_ - *H*_initial_)) for the fpSi and pSi-ch samples. The stability of the different silicon surfaces can be ranked by their initial rate of degradation, with the stabilities being in the order: freshly etched porous Si > chitosan-coated pSi.

By comparing the degradation kinetics of the porous silicon samples using normalized reflectance values (either rugate position or EOT) and normalized *H* parameter values, we conclude that it is possible to obtain semiquantitative information about porous silicon stability using color data. In contrast, using the hue of the as-acquired images to monitor complete degradation is limited due to the interfering effect of the reflection of the broad light source spectrum from the porous silicon, silicon substrate, and other surfaces within the light path. However, the use of a different light source with increased intensity in the blue-green regions of the spectrum compared to the lamp used may reduce this problem. The behavior of the hue parameter for porous rugate samples with the reflectance band at *λ* < 560 nm is also very dependent on the white balance value used during the image pre-processing step.

## Conclusions

We have demonstrated that the degradation of porous silicon in basic aqueous buffers can be monitored in situ by digital imaging with a consumer-grade digital camera and have validated this approach with simultaneous spectrophotometric measurement of the optical reflectance spectra. An approximately linear correlation between the wavelength of the maximum of the rugate reflectance band and an *H* parameter derived from the HSV color space was observed during the degradation process. A similar relationship was also noted between the *H* parameter and the effective optical thickness of the samples. These results indicate that the samples were degrading via dissolution of the pore walls, rather than just dissolution from the top of the porous silicon layer downwards.

The relative stabilities of the two porous silicon types obtained by the three measurement methods were consistent, indicating that all methods could be used to monitor relative sample degradation. However, whereas measurement of the rugate peak wavelength and effective optical thickness requires a spectrophotometer, the determination of the *H* parameter just required a consumer-grade digital camera and standard software. Indeed, the *H*-parameter approach could be applied using a low-cost camera or the camera within a mobile phone or mobile computing device. This would then allow such measurements to be made outside the laboratory and at comparatively low cost.

While this paper reports results from monitoring degradation of intact porous silicon films attached to a crystalline silicon substrate, a similar approach should be possible to monitor particles of porous silicon. The potential use of color measurements to monitor both degradation and drug delivery from porous silicon micro-particles would require only simple cameras and illuminants and could even be coupled to use with smartphones.

## Competing interests

MJS has financial ties to the following companies who may or may not benefit from the research presented here: Spinnaker Biosciences, TruTags, Pacific Integrated Energy, and Silicium Energy.

## Authors’ contributions

The study conception and design was carried out by MJS, MAA, and AN. The initial design of the image acquisition equipment was performed by GM, MAA, and MJS. MAA carried out the acquisition of the data. The analysis and interpretation of the data was performed by MAA, LFCV, and GM. The preparation of the manuscript was performed by LFCV, GM, MAA, and ASC. The critical revision was performed by GM and MJS. All authors read and approved the final manuscript.
